# Trimethoprim-sulfamethoxazole induced circulatory shock in a human immunodeficiency virus uninfected patient: a case report and review

**DOI:** 10.1186/s40360-018-0269-3

**Published:** 2018-11-20

**Authors:** Patricia Liu, Gregory P. Ranches, Jeffrey A. Gold

**Affiliations:** 1 Department of Internal Medicine, Oregon Health & Science University, VA P-3-GP1, block 4, 3181 SW Sam Jackson Park Rd, Portland, OR 97239-3098 USA; 2 Division of Pulmonary and Critical Care Medicine, Oregon Health & Science University, Portland, Oregon USA

**Keywords:** Adverse drug reaction, Shock, HIV, CD4+ count, IL-6, Trimethoprim-sulfamethoxazole

## Abstract

**Background:**

Severe systemic reactions resembling septic shock have been described following trimethoprim-sulfamethoxazole (TMP-SMX) administration. Nearly all cases described in the literature occurred in HIV-infected patients.

**Case presentation:**

We present a 42-year-old woman with a history of systemic lupus erythematosus (SLE) who was admitted to the Intensive Care Unit (ICU) twice with fever and circulatory shock after taking a dose of TMP-SMX 800–160 mg. She had no respiratory distress, urticarial rash or eosinophilia on presentation. Infectious workup during both admissions was negative and treatment with antibiotics, steroids and vasopressors was de-escalated with clinical improvement. She was found to be HIV negative, however, labs revealed a low CD4+ count.

**Conclusions:**

TMP-SMX can rarely result in a severe, non-anaphylactic circulatory shock; if initially unrecognized, patients may undergo repeat drug exposure with an associated high morbidity risk. While more commonly reported in HIV individuals, this case demonstrates that TMP-SMX related circulatory shock can occur in a HIV negative patient.

## Background

Adverse drug reactions to antibiotics complicate the management of any infection. Trimethoprim-sulfamethoxazole (TMP-SMX) is generally well tolerated in non-human immunodeficiency virus (HIV) infected patients, however serious adverse reactions occur at an approximate rate of 3–5% [1]. More recently, severe systemic reactions resembling septic shock have been described following TMP-SMX administration [2–4]. Nearly all cases described in the literature occurred in HIV-infected patients, with the exception of two HIV-negative cases [5]. Here we report a case of a HIV-negative patient who twice developed circulatory shock following exposure to TMP-SMX.

## Case presentation

A 42-year-old woman with history of SLE on hydroxychloroquine, mycophenolate and prednisone, complicated by pancytopenia, presented with severe back pain 30 min after taking oral TMP-SMX 800-160 mg for paronychia. She was found to be febrile to 39.1 °C, hypotensive at 88/63 mmHg, and tachycardic at 107 BPM. Laboratory testing revealed a white blood cell (WBC) count of 8.63 × 10^3^/uL (97.3% neutrophils, 0.1% eosinophils, 0.2% lymphocytes, with a baseline WBC of 2 × 10^3^/uL), lactate 2.3 mmol/L and creatinine of 1.3 mg/dL (baseline 0.7 mg/dL) (Table 1). HIV ELISA was negative but the CD4+ count was low at 64 cells/uL. Computed tomography (CT) angiogram of the chest showed no evidence of pulmonary embolus or infection and urinalysis was not suggestive of infection. Physical exam was significant for diffusely erythematous and warm skin without macules, papules, or urticaria. There were no other focal findings on physical exam. The patient was initially managed with aggressive 80 mL/kg intravenous (IV) fluid resuscitation, norepinephrine, broad-spectrum antibiotics (IV vancomycin and IV piperacillin-tazobactam), and stress dose steroids (hydrocortisone 50 mg IV every 6 h). Her hypotension resolved quickly over the first 36 h of admission. Over the following 3 days blood and urine cultures remained negative and steroids, vasopressors and antibiotics were discontinued without recurrence of hypotension. Additional infectious work up, including hepatitis B, hepatitis C and toxoplasma, were negative. The patient was discharged home on hospital day four with pneumocystis jirovecii pneumonia (PJP) prophylaxis with atovaquone.

**Table 1:** Timeline of Presentations and Presenting Vitals and Labs

	May 2017	July 2016
Vitals		
Blood Pressure (mmHg)	88/63	79/45
Heart Rate (BPM)	107	121
Temperature (°C)	39.1	39.0
Respiratory Rate	22	23
O_2_ Saturation	96% on Room Air	97% on Room Air
Labs		
Basic Chemistry Panel	(baseline Cr: 0.7)	
Complete Blood Count	97.3% neutrophils 0.1% eosinophils 0.2% lymphocytes (baseline WBC: 2 × 10^3^/uL)	95.5% neutrophils 0.6% lymphocytes 0.5% eosinophils
Lactate	2.3 mmol/L	1.2 mmol/L
HIV Elisa	Negative	Not checked
CD4+ Count	64 cells/uL	Not checked
Infectious Work Up	CTA chest Urine cultures Blood cultures	CT head CT cervical, thoracic and lumbar spine CT abdomen and pelvis Echocardiogram Urine cultures Blood cultures

Ten months prior, the patient had a similar presentation several hours after taking her second dose of oral TMP-SMX 800-160 mg, which was prescribed for an abscess of the mons pubis. On presentation, she was hypotensive, tachycardic and febrile. Laboratory testing revealed a WBC of 10.9 × 10^3^/uL (95.5% neutrophils, 0.6% lymphocytes and 0.5% eosinophils), acute kidney injury (creatinine 1.1 mg/dL) and lactate of 1.2 mmol/L. She was treated similarly for presumed septic shock. Work-up for infectious etiologies including blood and urine cultures, CT imaging of head, spine and abdomen as well as an echocardiogram was unrevealing. She was weaned off vasopressor support within 24 h. Her steroids and antibiotics were discontinued within 72 h with clinical improvement and she was discharged home with the diagnosis of septic shock with unknown etiology.

## Discussion and conclusions

TMP-SMX causes serious adverse drug reactions (ADR) in 40–65% of HIV-infected persons and only 3–5% in non-infected persons [6–9]. Previous studies demonstrated that rates of ADRs correlate with HIV viral load and that the highest rates of some ADRs occur when CD4+ counts fall below 100 cells/uL [10, 11]. More recently, severe systemic reactions resembling septic shock have been described following TMP-SMX administration. To our knowledge, this is the first reported case of a severe systemic reaction to TMP-SMX in an HIV-negative, CD4+ lymphocytopenic patient. Although HIV viral load has historically been the predominant risk factor, our case raises the question of whether low CD4+ cell count, which can occur independent of HIV in connective tissue diseases or due to an adverse effect of immunosuppressive medications, could be associated with TMP-SMX induced circulatory shock.

Other etiologies of the patient’s shock were considered, such as sepsis, adrenal insufficiency and anaphylaxis. Although she was prescribed TMP-SMX for an infection both times (mons pubis abscess and paronychia), they were minor infections. Other data against septic shock include an otherwise negative infectious work up, negative lactate and rapid resolution of shock. She was given stress dose steroids given the extent of her hypotension but they were quickly tapered without recurrence of shock, making adrenal insufficiency unlikely. Lastly, she did not have urticaria, eosinophilia, laryngeal edema or bronchospasms that are typical for anaphylactic shock. While TMP-SMX has many important drug-drug interactions to consider, there are no established reactions between TMP-SMX and the patient’s medication list to explain this reaction.

We identified 17 cases in the literature of TMP-SMX induced circulatory shock similar to that noted in our patient (Table 2) [2–5, 12–14]. Fifteen of the 17 cases were in HIV+ patients. Including the case presented here, all 18 patients experienced an abrupt onset of fever and hypotension within minutes to hours after the administration of TMP-SMX. Ten of the 18 patients were noted to have a rash; nine of which were described as diffusely erythematous and one that was described as urticarial. In terms of end organ damage, it appears that acute kidney injury is the most common, occurring in 9 of the 18 patients. Hypoxemia, pulmonary infiltrates and elevated liver enzymes were also reported in some cases.

**Table 2:** Demographics and clinical characteristics of trimethoprim-sulfamethoxazole-induced severe reactions in the literature

Author, year	Age (yrs.)	HIV Status	CD4+ (cells/mm^3^)	TMP-SMX Regimen	Time of Onset	Fever (°C)	Hypotension (mmHg)	Rash	Hypoxemia	Pulm. Infiltrates	Acute Kidney Injury	Elevated liver enzymes
Johnson et al. 1990	28	AIDS	NS	DS tablet	Several Hours	39.0	SBP 90	–	+	–	–	–
	38	HIV (+)	NS	DS tablet	Several Hours	39.0	SBP 75	–	–	–	–	–
Kelly et al. 1992	46	AIDS	NS	DS tablet	< 4 h	39.8	60/0	–	+	+	+	+
	34	AIDS	NS	DS tablet	< 1 h	39.4	80/40	+	+	+	+	+
	22	AIDS	NS	DS tablet	4 h	40.2	70/0	–	–	–	+	+
	31	AIDS	NS	SS tablet	1 h	40.2	+	+	NS	NS	NS	NS
	46	AIDS	NS	? IV dose	Several minutes	40.0	60/40	+	+	+	NS	NS
	28	AIDS	NS	? PO dose	30 min	40.0	70/40	+	+	+	NS	NS
	34	AIDS	NS	DS tablet	30 min	41.0	60/28	+	+	+	+	NS
	23	AIDS	NS	? PO dose	“quickly”	40.5	82/0	+	+	+	NS	NS
	28	HIV (+)	NS	DS tablet	< 2 h	39.0	65/0	+	+	+	NS	NS
	38	HIV (+)	NS	NS	NS	39.0	75/0	+	NS	+	+	+
Nguyen et al. 1993	37	HIV (+)	190	DS tablet	2 h	40.0	84/30	–	–	–	+	–
Caumes et al. 1996	48	AIDS	366	? PO dose	20 min	+	+	+	–	–	–	–
Scourfield et al. 2010	38	HIV (+)	13	? PO dose	1 h	40.0	75/34	–	NS	+	NS	NS
Persichino et al. 2016	36	HIV (+)	24	DS tablet	1 h	40.1	72/23	–	–	–	+	–
Abbas et al. 2017	85	HIV (−)	NS	? PO dose	5 h	+	NS	–	–	–	+	–
	85	HIV (−)	NS	? PO dose	NS	NS	NS	+	–	–	–	–
Presented Case	42	HIV (−)	64	DS tablet	< 2 h	39.1	88/63	+	–	–	+	–

-: Negative Finding; +: Positive Finding; *NS* Not Stated, *SBP* Systolic Blood Pressure

The mechanism behind TMP-SMX induced circulatory shock remains unclear. At present, whether this reaction represents a form of immune activation, cellular toxicity or non-specific immune reaction is unclear. Nguyen and colleagues have argued that TMP-SMX induced shock is likely not a traditional anaphylactic reaction based on the lack of TNF-α and IgE elevation upon re-challenge of a HIV+ patient with known TMP-SMX induced shock [12]. Instead they observed a marked rise and fall of IL-6 temporally associated with the episode of shock. Interestingly, IL-6 has also been implicated in TMP-SMX induced aseptic meningitis and macrophage activation syndrome [15–17]. As IL-6 is also known to be elevated in septic shock associated with bacterial infections, one hypothesis to consider is whether TMP-SMX induced shock and septic shock share a final downstream mechanism [18]. Further investigation into the cytokine profile and cellular responses involved in TMP-SMX induced shock in warranted.

It is also not clear whether trimethoprim or sulfamethoxazole, or both, are drivers of the shock reaction; many hypotheses exist. Some in vitro studies suggest that incubation of the hydroxylamine of sulfamethoxazole with HIV-infected cells produced concentration-dependent toxicity that is significantly greater than in non-HIV infected cells [10]. This is one possible explanation for the increased rates of serious sulfonamide adverse drug reactions among patients with HIV. Trimethoprim, an inhibitor of dihydrofolate reductase, has been proposed to cause aseptic meningitis [19]. One study observed higher in vitro IL-6 production in cells of patients with trimethoprim induced systemic adverse reactions compared to trimethoprim tolerant patients [16]. Trimethoprim can also inhibit renal tubular secretion of potassium and creatinine, thus leading to adverse events such as hyperkalemia and acute tubular necrosis [20]. However, to date, there are no studies that implicate trimethoprim alone in the development of hypotension or shock.

There have been few case reports and series describing shock in reaction to TMP-SMX, overall, cases may be under reported or lack complete clinical datasets. To our knowledge, this case is the third described in a patient who is HIV negative. TMP-SMX induced shock is not a widely recognized phenomenon, but is particularly important to consider in lymphopenic patients with circulatory shock and recent TMP-SMX administration. Adding TMP-SMX to the allergy list in these patients is of utmost importance in order to prevent future recurrences.

## Abbreviations

ADR: Adverse Drug Reaction
CT: Computed Tomography
ELISA: Enzyme Linked Immunosorbent Assay
HIV: Human Immunodeficiency Virus
ICU: Intensive Care Unite
IL: Interleukin
PJP: Pneumocystis Jirovecii Pneumonia
SLE: Systemic Lupus Erythematous
TMP-SMX: Trimethoprim-sulfamethoxazole
TNF: Tumor Necrosis Factor
WBC: White Blood Cell
